# Clinical utility of the low-density Infinium QC genotyping Array in a genomics-based diagnostics laboratory

**DOI:** 10.1186/s12920-017-0297-7

**Published:** 2017-10-06

**Authors:** Petr Ponomarenko, Alex Ryutov, Dennis T. Maglinte, Ancha Baranova, Tatiana V. Tatarinova, Xiaowu Gai

**Affiliations:** 1 Department of Biology, University of La Verne, La Verne, CA USA; 2 Center for Personalized Medicine, Department of Pathology and Laboratory Medicine, Children’s Hospital Los Angeles, Los Angeles, CA USA; 3 School of Systems Biology, George Mason University, Fairfax, VA USA; 4 Research Center for Medical Genetics, Moscow, Russia; 5 Atlas Biomed Group, Moscow, Russia; 6 Department of Pathology and Laboratory Medicine, USC Keck School of Medicine, Los Angeles, CA USA

**Keywords:** Quality control, Infinium QC Array-24, Ethnicity, Sample identity, Kinship, Clinical exome sequencing, NGS-based molecular diagnostic tests

## Abstract

**Background:**

With 15,949 markers, the low-density Infinium QC Array-24 BeadChip enables linkage analysis, HLA haplotyping, fingerprinting, ethnicity determination, mitochondrial genome variations, blood groups and pharmacogenomics. It represents an attractive independent QC option for NGS-based diagnostic laboratories, and provides cost-efficient means for determining gender, ethnic ancestry, and sample kinships, that are important for data interpretation of NGS-based genetic tests.

**Methods:**

We evaluated accuracy and reproducibility of Infinium QC genotyping calls by comparing them with genotyping data of the same samples from other genotyping platforms, whole genome/exome sequencing. Accuracy and robustness of determining gender, provenance, and kinships were assessed.

**Results:**

Concordance of genotype calls between Infinium QC and other platforms was above 99%. Here we show that the chip’s ancestry informative markers are sufficient for ethnicity determination at continental and sometimes subcontinental levels, with assignment accuracy varying with the coverage for a particular region and ethnic groups. Mean accuracies of provenance prediction at a regional level were varied from 81% for Asia, to 89% for Americas, 86% for Africa, 97% for Oceania, 98% for Europe, and 100% for India. Mean accuracy of ethnicity assignment predictions was 63%. Pairwise concordances of AFR samples with the samples from any other super populations were the lowest (0.39–0.43), while the concordances within the same population were relatively high (0.55–0.61). For all populations except African, cross-population comparisons were similar in their concordance ranges to the range of within-population concordances (0.54–0.57). Gender determination was correct in all tested cases.

**Conclusions:**

Our results indicate that the Infinium QC Array-24 chip is suitable for cost-efficient, independent QC assaying in the settings of an NGS-based molecular diagnostic laboratory; hence, we recommend its integration into the standard laboratory workflow. Low-density chips can provide sample-specific measures for variant call accuracy, prevent sample mix-ups, validate self-reported ethnicities, and detect consanguineous cases. Integration of low-density chips into QC procedures aids proper interpretation of candidate sequence variants. To enhance utility of this low-density chip, we recommend expansion of ADME and mitochondrial markers. Inexpensive Infinium-like low-density human chips have a potential to become a “Swiss army knife” among genotyping assays suitable for many applications requiring high-throughput assays.

**Electronic supplementary material:**

The online version of this article (10.1186/s12920-017-0297-7) contains supplementary material, which is available to authorized users.

## Background

The costs of NGS-based tests could be significant, the experimental workflow could be very complex, the number of steps and people involved could be high, the amount of data is large, and the consequences of errors such as sample mix-ups hence misdiagnosis could be severe. In their clinical laboratory standards for next-generation sequencing, the American College of Medical Genetics and Genomics (ACMG) emphasizes the essentiality of QC measures for identification of failed sequencing runs, but also for tracking identities of each sample throughout the testing process. To facilitate the QC, the development of a cost-efficient, independent genotyping assay is paramount [1].

Infinium QC Array-24 chip (Infinium QC) contains 15,949 markers, divided into eight categories (Table 1 and Additional file 1). Some of the markers are associated with easily identifiable traits such as hair color, eye color, sex, and blood type. Hence, this low-density chip allows cross-referencing with known sample metadata and, therefore, confirmation of sample identity prior to downstream processing. Other Infinium QC biomarkers are associated with certain traits and ethnicities, ADME responses or tissue compatibility. Collectively, these markers allow genetic stratification of samples. In addition, the array also covers significant portions of the Y chromosome, allowing for identification of its mosaic loss, previously shown to contribute to many clinical conditions including cancer and Alzheimer disease.

**Table 1:** Infinium QC Array-24 variants sorted by their category and source

Marker Category	Category Description	Number of Markers
ADME	Pharmacogenomics, from PharmADME.org	1009
AIM	Ancestry Informative markers from exome array (http://genome.sph.umich.edu/wiki/Exome_Chip_Design#Ancestry_Informative_Markers)	2910
Blood group	From NCBI’s *dbRBC* database covering 51 blood group defining genes http://www.ncbi.nlm.nih.gov/projects/gv/rbc/xslcgi.fcgi?cmd=bgmut/systems	1659
Fingerprint	High MAF SNPs unlikely to be in LD with each other, from http://www.cstl.nist.gov/strbase/SNP.htm and http://alfred.med.yale.edu/alfred/index.asp	477
Linkage	Linkage Panel by Illumina, contains heterozygous SNPs to test for Mendelian disorders, from Linkage 12 array	5486
Extended MHC	Variants from extended major histocompatibility complex MHC covering 8 Mb region containing immune markers	930
Mitochondrial	Determination of mtDNA haplogroups	141
Sex chromosomes	X-chromosome specific	1840
	Y-chromosome specific	1401
	Pseudoautosomal Regions	535

We explored potential applications of this low-cost Infinium QC array in the studies of human specimens, including verification of the identity of human biomaterial, determination of its ethnic origin, and evaluation of the accuracy of sample specific variant calling. In this study, we first compared the genotyping results of this low-cost Infinium QC assays with substantially more expensive Whole-Exome Sequencing/Whole-Genome Sequencing (WES/WGS) data. Second, we compared genotype calls by the Infinium QC array to those by other sequencing or genotyping platforms, in particular, with 1000 Genomes WGS, Illumina’s Infinium Omni 2.5 and Affymetrix’s Genome-Wide Human SNP Array 6.0 microarray chips. Third, we assessed the power and accuracy of ethnicity determination using the 2000 ancestry informative markers included on the array based on Infinium QC data of 664 individuals studied by the 1000 Genomes Project, as well as Infinium QC equivalent data of 645 individuals studied by the National Genographic Project. Fourth, we determine the concordance rate of the Infinium QC genotyping calls with variant calls from WES data of 35 of our own patient samples. Fifth, we determined the Infinium QC chip’s ability to determine kinships and to discriminate self-self, parent-child, siblings, second-order relatedness, and totally unrelated individuals using the 1000 Genomes Project data and our own patient data. Results of these analyses strongly support utility of this low-density array in a molecular diagnostic laboratory.

## Methods

### Materials

Human QC manifests and test data were downloaded from Illumina website (http://support.illumina.com/array/array_kits/infinium-qc-array-kit/downloads.html). It contains genotyping data of 15,949 markers at 15,837 unique chromosome positions from 664 individuals.

Affymetrix 6.0. (AFFY) and Illumina’s Omni 2.5 (OMNI) data were downloaded from EBI (ftp://ftp.1000genomes.ebi.ac.uk/vol1/ftp/release/20130502/supporting/hd_genotype_chip) for individuals with pedigree matching the rest of the population by admixture vector. OMNI data includes genotypes of 2,458,861 chromosomal loci and 2318 individuals. AFFY data contains genotypes of 905,788 chromosomal positions and 3450 individuals.

1000 Genomes Project (1KG) dataset was downloaded from EBI (ftp://ftp.1000genomes.ebi.ac.uk/vol1/ftp/release/20130502) and for related individuals from (ftp://ftp.1000genomes.ebi.ac.uk/vol1/ftp/release/20130502/supporting/related_samples_vcf **).** It contains genotypes of 2504 individuals merged from multiple sets of genotyping and NGS data experiments, and is considered a gold standard. The family information was extracted from the pedigree file available on the 1000 Genomes website (ftp://ftp.1000genomes.ebi.ac.uk/vol1/ftp/technical/working /20130606_sample_info/20130606_g1k.ped) [2].

Reference dataset for GPS and reAdmix [3] was obtained from the supplemental data to Elhaik el al. (2014) [4]. In order to enable comparison with this data, sets of individual SNPs were converted to the 9-dimensional admixture vectors (“North East Asian”, “Mediterranean”, “South African”, “South West Asian”, “Native American”, “Oceanian”, “South East Asian”, “Northern European”, “Sub-Saharan African”) using the ADMIXTURE software [5, 6] in the supervised mode. Genotypes for 1000 Genomes Project dataset were obtained from http://www.1000genomes.org/category/population/ [2].

There were 48 additional DNA samples genotyped on Infinium QC array at the Center of Personalized Medicine, Children’s Hospital Los Angeles. These were de-identified DNA samples from CHLA patients; 33 of the samples were used for validation of our Clinical Exome Sequencing (CES) test. They are stored at the CHLA Pediatric Research Biorepository, which has granted the institutional waiver of consent for research purposes. Furthermore, the patients have granted us the permission to share their anonymized data using the patient consent form.

### Methods

#### Data preparation and organization

The genotyping data and manifest files were stored and analyzed in a custom Oracle database. To extract individuals and positions for comparison, we used VCFtools v0.1.13 (https://vcftools.github.io/index.html) [7]; this software was also used for sorting and merging the variant calling (vcf) files from genotyping experiments (e.g. Illumina’s Infinium Omni 2.5 and Affymetrix’s Genome-Wide Human SNP Array 6.0 microarray chips), as well as 1000 Genomes data. The vcf files were converted to the binary plink format (*bim*, *bed*, and *fam* files) using PLINK v1.90b3d (https://www.cog-genomics.org/plink2) [7, 8]. PLINK was also used to filter out tri- and quadri-allelic SNPs for within and between datasets comparisons, to calculate concordance for a subset of non-missing markers and to extract all discordant markers.

We first extracted genotyping calls of 664 individuals from the Infinium QC, the OMNI, and the AFFY arrays at shared marker positions using VCFtools [9]. In-house scripts and pipelines were utilized for file manipulation, analysis of concordance rates, identification of discordant markers and for evaluating the ability to uniquely identify samples. After excluding the multi-allelic positions, Infinium QC array data and AFFY, OMNI and 1KG datasets were compared using PLINK for all 664 individuals, also present in AFFY, OMNI and 1KG data.

Essentially same procedure was used for comparing Infinium QC data with our in-house WES variant calls. The regions for comparison were selected according to the exome design file prepared using the entire *refGene* table (http://refgene.com) based on the hg19 genome assembly, which was downloaded from the UCSC Genome Browser using the Table Browser [10]. Next, a BED track of all coding exons extended by 5 bp in each direction was downloaded using the Table Browser. The RefSeq transcript identifier in the BED file was mapped to its gene symbol in the *refGene* table. Exons duplicated across multiple transcripts of the same gene were removed to ensure that each exon was represented only once. The records within the resulting file were sorted by their genomic locations.

To assess suitability of the Infinium QC beadchip for determination of sample identity, the concordances of genotype calls and allele calls between every possible pair of individuals were calculated using in-house C++ programs and compared with the output of PLINK.

Custom C++ applications were written specifically for this project, and are available upon request. Additionally, selected tools from the PLINK (http://pngu.mgh.harvard.edu/~purcell/plink/) and SAMTools (http://github.com/samtools/samtools) [11] packages were employed.

#### Concordance calculation

The concordances of variant calls between the Infinium QC chip and other platforms were calculated after following filtering steps:

- Only bi-allelic variants were used for the calculation, while tri-allelic and other multi-allelic variants were filtered out
- Y chromosome variants were analyzed separately, since the call rates for the males were consistently lower than for females

When comparing any two platforms, the concordance was assessed for genotype calls at all shared marker positions. Exactly matching genotypes were recorded as concordant. For each sample, overall concordance was reported as a ratio of all concordant genotype calls to the number of shared marker position with genotypes called in both datasets: “Number of Concordant positions”/“Number of Common positions.”

### Sample identification

To test the ability of the Infinium QC array to detect sample swaps, all possible sample mix-ups were simulated at the different levels of relatedness between samples, including parent-child, siblings, family, population and “all human samples” and analyzed for concordance. Simulations were conducted using C++ software developed in-house. To test whether the separation of the distributions of “self-hits” vs. “mismatches” is significant, Kolmogorov-Smirnov statistics were used.

### Genotyping with Infinium QC array

Using the Infinium QC arrays on an iScan instrument, we genotyped 48 DNA samples in-house. Most of these samples were also used for the validation of our Clinical Exome Sequencing (CES) test. These samples were selected from a diverse set of patients with GPS-predicted [4] ethnicities spanning the globe: Finnish (*N* = 2), Bulgarian (*N* = 4), Vietnamese (N = 4), Japanese (*N* = 3), Hispanic (*N* = 7), Peruvian (*N* = 12), African American (N = 1), Lebanese (N = 1), Bermudian (N-1), and Kuwait (N = 2). CES data were processed using the bcbio pipeline v.0.9.6 (https://github.com/chapmanb/bcbio-nextgen).

### Infinium QC data analysis and preparation

Genotype calls were first made using the Illumina GenomeStudio software suite. To generate outputs in PED and MAP formats for downstream analysis in PLINK, a PLINK export plug-in was installed in GenomeStudio. “Chromosome 0” labeled control variants and indels were filtered out. To normalize the variants for which the bottom designation corresponded to the forward strand, we created lists of variants to be filtered out and flipped after matching to the records in “Strand Report” file provided for the Infinium QC Array. For all 48 Infinium QC samples, a binary PED file, and a VCF file containing variants were created using PLINK. The VCF file was compressed and indexed with the SAMtools utility tools tabix and bgzip (part of HTSlib-1.3.1 https://github.com/samtools/htslib) [11–13].

### Removing underperforming markers

We identified and excluded markers that were consistently discordant between different platforms across at least 10% of samples. We also removed from our analysis all Infinium QC variants that correspond to HLA genes, since HLA genes and the MHC region in general are known to be extremely complex with high sequence similarities between genes and hence unreliable genotyping calls [14]. The description of 319 excluded makers is in the Supplement.

### Provenance prediction

Ethnicity prediction was done with the ADMIXTURE tool [5, 6] in supervised mode. In brief, the genotype data were converted into K = 9 dimensional vectors, followed by GPS and reAdmix analyses. Both Geographic Population Structure (GPS) [4] and reAdmix [3] algorithms were used to infer the provenance of the samples and to confirm self-reported ethnic origin. For each tested individual, GPS algorithm determines a location on a world map, where people with similar genotypes are likely to reside. For individuals produced by recent ethnic mixing (i.e. children of parents from two different ethnic groups), GPS predictions were followed by analysis with reAdmix, which models an individual as a mix of populations and permits user-guided conditional optimization.

## Results

### Concordance of genotype calls between platforms

Infinium QC array is comprised of 15,949 markers covering 15,837 unique loci. Agreement between variant calls of the same sample using different experimental platforms provides information about the quality of the Infinium QC array. We therefore compared genotypes reported by the Infinium QC array with that of the 1000 Genomes Project WGS, Omni and Affymetrix genotyping arrays. Concordances of genotype calls between Infinium QC and OMNI, AFFY 6.0 and WGS were determined to be 99.63%, 99.66% and 99.39%, respectively, when only non-missing bi-allelic calls between two sets were compared. For the Y chromosome-specific comparison of Infinium QC and 1000 Genomes data, the concordance of calls was at 95.68%. Details of this analysis are provided in the supplementary materials (see Additional file 2: Tables S1-S4 and Figure S1).

The majority of discordant calls were consistent across all pairs of different datasets (see Additional file 2: Table S4). The Top 30 most discordant markers between Infinium QC and the 1000 Genomes WGS datasets were compared to OMNI and Affymetrix datasets. Most of the markers are present only on OMNI or Affymetrix platforms. Only four of these markers were discordant in both platform-specific comparisons. These four markers were also discordant between OMNI and Affymetrix results, indicating a likely common source of error.

We obtained from Illumina the Infinium QC data of 503 out of 664 individuals previously also studied by the 1000 Genomes Project, for whom the latest release of phase 3 was available. When comparing the Infinium QC data with the 1000 Genomes data, we found that markers were discordant in 0 to 489 samples. Therefore, we identified and excluded markers that were consistently discordant between platforms for at least 10% of samples (total 67 variants, including 4 HLA markers, listed in the Additional file 1). We recommend excluding these under-performing markers for further analysis, as we did in current study. We have also removed all Infinium QC variants that fall into the HLA genes, since their calls were previously shown to be unreliable [14]. Overall, 319 markers were excluded.

### Utility of Infinium QC for asserting identity of a human sample

**Fig. 1:**
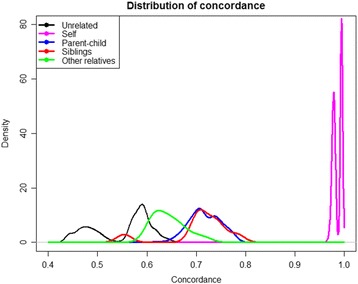
Concordance for same samples between Infinium QC and 1000 Genomes data (*purple*), and for different unrelated samples (*black*), between parent and child, siblings, and other relatives

**Fig. 2:**
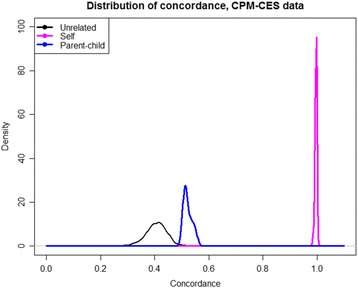
Concordance histogram for all possible pairs of samples from iScan and CES experiments

To investigate the utility of Infinium QC for identifying human samples and possible sample mix-ups, the concordance values for different samples and different platforms were calculated for all possible pairs of samples, either matched or purposefully mismatched, and every pair of platforms. Distributions of resultant concordance values shown at Figs. 1 and 2, including the concordance between matched and purposefully mismatched for simulation of accidental sample swaps on parent-child, sibling, family, and population-wide datasets. For matched and mismatched sample pairs, the distributions of concordance rates were significantly different and separated well. Kolmogorov-Smirnov statistic values are shown in the Additional file 3.

### Concordance analysis for related individuals

The pairwise sample concordance estimation is a powerful tool to evaluate genetic similarity between family members, relatives and general populations. To assess this, we analyzed a set of 35 samples, including three family trios, for which we obtained both the Infinium QC and clinical whole-exome sequencing data. The concordance histogram for the calls in samples with varied degrees of relatedness is presented in Fig. 2.

### Kinship calculation

The kinship coefficient and identity by descent (IBD) were evaluated using the KING [15] software (Table 2). Kinship coefficients discriminate between various degrees of relationship, while failing to distinguish between “Child-parent” and “Siblings” (see Additional file 2: Table S5 for theoretical values). These types of sampling pairs were resolved using IBD [16], which highlights if a DNA fragment is a copy of a single piece of DNA of some ancestral individual. According to recommendations of the authors of KING, in order to distinguish between parent–child from relationships, one needs to “examine the observed IBS making use of the fact that IBS between a parent–offspring pair is always 1 or 2 at any SNP in the absence of genotyping errors” [15]. To determine the relatedness cut-offs, we use 2208 pairs of individuals genotyped on the Illumina Omni platform. Two of the recorded pairs of siblings in 1000 Genomes database (NA20334/NA20344 and NA20336/NA20344) have suspiciously weak similarity (kinship of 0. 0148 and −0.0081), while the pair NA20334/NA20336 have kinship consistent with siblings (0.2251). See (http://www.internationalgenome.org/data-portal/sample/NA20344).

**Table 2:** Kinship (estimated by KING) 1000 Genomes

Relatedness	Median Kinship	Sample size	Theoretical kinship	Min KIN	Max KIN
Siblings	0.2354	9	0.25	−0.0081	0.3029
Parent-Child	0.2441	221	0.25	0.1712	0.2620
Second Order	0.1107	9	0.125–0.1875	0.0714	0.1475
Unrelated	−0.1300	1679	<0.001	−0.3074	0.0443

Experimentation with 1000 Genomes data prompts us to recommend utilization of Infinium QC chip for discerning the degree of relatedness between individuals within the study set. As the first step of stratification, kinship coefficients are calculated; as the “parent-child” and “siblings” subgroups are discriminated based on the joint assessment of kingship coefficient and IBD. IBS0 for the “Parent-child” ranges between 0 and 0.0157, median at 0.0008. IBS0 for the “Siblings” ranges between 0.0114 and 0.0215, median at 0.0167. Hence, there division between “Siblings” and “Parent-child” IBS0 is not a sharp one. More sensitive methods of relatedness analysis are being developed (see, for example Genetic Relationship and Fingerprinting (GRAF) tool [17]).

### Ethnicity prediction

SNP array data for populations sampled in this study was compared to the worldwide collection of populations based on Illumina’s Geno 2.0130 K ancestry-informative markers (AIM) [18]. Infinium QC includes a subset of these markers (*N* = 1897). As it was demonstrated earlier [4], when the number of AIMs used to determine admixture vectors is reduced to 500, the difference between the admixture vectors obtained from the complete set of AIMs and the reduced set does not exceed 6%, which is within the natural variation range of populations grouped by sub-continents.

We used admixture vectors obtained from running ADMIXTURE software in supervised mode with reference dataset from Elhaik et al. (2014) [4].

Next, we used GPS [4] and reAdmix [3] algorithms to infer provenance of the samples and to confirm their self-reported ethnic origin. For each tested individual, GPS algorithm determines its provenance - a location on a world map, where people with similar genotypes are likely to reside; GPS is not suitable for analysis of recently mixed individuals, for example, these with parents from two different ethnic groups. In such case, GPS reports high degree of uncertainty in prediction. To address this issue, reAdmix algorithm represents an admixed individual as a weighted sum of reference populations.

### GPS analysis

To validate Infinium QC as a genotyping method for deriving the provenance of the sample, we applied GPS to 645 individuals previously analyzed by the Geno 2.0 chip in frame of the National Genographic Project [4, 18]. After extracting only SNPs overlapping in Geno 2.0 and Infinium QC (1897), the samples were analyzed using ADMIXTURE in the supervised mode for K = 9.

Depending on provenance of a particular sample, GPS accuracy varies as it primarily depends on the extent of coverage of a particular geographical region and ethnic group in available databases. By limiting GPS algorithm to Infinium QC markers only, and utilizing leave-one-out approach, we showed that the LD chip based assessment of sample provenance worked predominantly at the subcontinental level. At the level of population, median accuracy of GPS-based provenance prediction was at 67%, and mean accuracy was at 63%. Among the groups with at least 10 samples per population, the highest accuracy (9 out of 10) was for inhabitants of the Vanuatu. This result is not surprising since it is a predominantly rural population confined to an archipelago. The only misclassified Vanuatu individual ended up in the related “Papua New Guinea, coastal” category. Another group with high prediction accuracy was Sardinian, with 12 correct population-level assignments out of 15, with three misclassified individuals being assigned to geographically proximal “Bulgarian”, “Iberian” and “Lebanese” groups. As relatively isolated Sardinian population retained its genetic similarity to Neolithic farmers (such as Otzi) to substantially larger degree than other Europeans [19], the high accuracy achieved for this group is not surprising as well. At the other end of the spectrum we observed Gujaratis, with only 1 out of 12 correct predictions. In this population, all incorrect predictions assigned to other Indian groups. It is important to note that National Geographic Reference population of Gujarati Indians was assembled from specimens collected in Houston, Texas, from individuals self-reported as Gujarati, and previously shown to be admixed [20]. Another group with poor inference of the provenance, the Bulgarians, with 1 out of 15 correct predictions, has been incorrectly scattered among geographically proximal “German”, “Greek”, “Ingush”, “Italian”, “Romanian”, “Russian”, and “Sardinian” groups. It should be noted that Bulgarian demonstrates the most significant admixture among Slavs in the South of Europe [21], and shows the highest diversity of haplogroups [22]. Notably, “Bulgarian” provenance was difficult to discern even with the larger Geno 2.0 chip, with the accuracy of predictions reaching only 50% [4]. Therefore, we conclude that even in the worst-case scenario with a majority of samples mis-assigned at population level, the Infinium QC chip successfully sorts out the provenance of the samples at subcontinental scale of super-populations. See supplement for details (Additional file 2: Tables S6-S7 and Figures S2-S6, plots generated with the Plotly Online Chart Maker, plot.ly).

Next, we obtained from Illumina Infinium QC genotyping data of 664 individuals that were also studied by the 1000 Genomes Project. Table 3 shows is the description of ethnic composition of the samples in that dataset.

**Table 3:** Ethnic composition of the subset of the 1000 Genomes samples genotyped on the Infinium QC array

Population code	Number of samples	Population
ASW	90	Americans of African Ancestry in SW USA
CEU	88	Utah Residents (CEPH) with Northern and Western Ancestry
CHB	38	Han Chinese in Beijing, China
GIH	77	Gujarati Indian from Houston, Texas
JPT	45	Japanese in Tokyo, Japan
MXL	82	Mexican Ancestry from Los Angeles, USA
PUR	72	Puerto Ricans from Puerto Rico
TSI	83	Toscani in Italy
YRI	88	Yoruba in Ibadan, Nigeria

In this study set, we have approximately equal numbers of samples (80–90) of NE, SE, AFR, SEA, EAS origins, plus admixed populations of Africans, Mexicans and Puerto-Ricans. Importantly, in this study set, non-admixed South American lineages were not represented. Among the Americans of African Ancestry in SW USA, 52% were mapped to Bermuda (where the ethnic mix resembles that of African Americans with 54% Black, 31% White, 8% Multiracial, 4% Asian, and 3% other); 41% was assigned to various countries in Africa, 6% to Puerto-Rico and one individual predicted to be East Greenlander. This individual, with reported ¾ African American grandparental ancestry, had non-African admixture vector, with predominant Native American component of 45%, followed by Northern European component of 24%. Ninety-seven percent of Utah resident samples ended up as mapped to various countries in Europe, with 74% mapped to Western and Northern Europe. Among the samples with self-reported Chinese origins, 55% got assigned as Chinese, and 45% as Japanese, while among the Japanese, 73% were identified as Japanese, and 27% as Chinese. This difference may be due to higher diversity of Chinese populations as compared to Japanese ones. Among the Gujarati Indian samples collected in Houston, Texas, 96% mapped to various locations in India and 4% to Pakistan. Among Mexicans, 67% of samples were mapped to Peru, 18% to Hidalgo Mexico, 10% to Puerto Rico and 4% to Mediterranean region. The latter observation is likely to reflect deficiency of the reference databases with respect to Mexicans, as well as the diversity and the admixture of Mexican population in Los Angeles. Among the Puerto Ricans, 43% were labeled as Puerto-Ricans, 15% as Africans, 7% as Bermudian, 1% as Peruvian, and the rest as Europeans. Italians (TSI) samples were predominantly mapped to Italy (46%), with 5% to Caucasus, 18% to other Mediterranean (Greece and Cyprus), and the rest to various countries in Europe. The LD chip correctly identified 94% of Yoruban samples, with 6% assigned to Kaokoveld Namibia.

From the two analyses presented above, we can conclude that Infinium QC chip is sufficient to provide continent-level resolution (Europe and Africa), while for some populations, such as Indians, it achieves the resolution at the sub-continental level. This is critically important for interpreting the likely pathogenicity of candidate variants as they may have different allele frequencies in different ethnic groups.

### reAdmix analysis

For each individual from the subset of 1000 Genomes database, we applied reAdmix algorithm, which represents a person as a weighted sum of modern populations represented as admixture vectors (Tables 4 and 5). As expected, historically admixed populations, for example, Puerto-Ricans are represented by the largest number of populations (1.78, on average), with the respective value of the most significant population being the smallest (0.59).

**Table 4:** reAdmix assignments, average number of ethnicities

Population	Average number of ethnic assignments per individual	Weight of the most significant ethnic assignment
PUR	1.78	0.59
CEU	1.58	0.67
MXL	1.39	0.65
ASW	1.28	0.76
TSI	1.25	0.74
GIH	1.18	0.82
CHB	1.13	0.90
YRI	1.01	0.99

**Table 5:** reAdmix assignments, grouped by 1000 Genomes categories

1000 Genomes	Global	Number of assignments	Total	Fraction
ASW	AFRICA	86	90	0.955556
ASW	NATIVE AMERICAN	2	90	0.022222
ASW	EUROPE	1	90	0.011111
ASW	MIX AFRICAN/EUROPEAN	1	90	0.011111
CEU	EUROPE	83	88	0.943182
CEU	NORTH ASIA	3	88	0.034091
CEU	NATIVE AMERICAN	2	88	0.022727
CHB	EAST ASIA	68	84	0.809524
CHB	INDIA	14	84	0.166667
CHB	NORTH ASIA	2	84	0.02381
GIH	INDIA	77	77	1
MXL	NATIVE AMERICAN	64	82	0.780488
MXL	EUROPE	9	82	0.109756
MXL	NORTH ASIA	6	82	0.073171
MXL	NEAR EAST	3	82	0.036585
PUR	NEAR EAST	17	72	0.236111
PUR	EUROPE	18	72	0.25
PUR	NATIVE AMERICAN	12	72	0.166667
PUR	AFRICA	12	72	0.166667
PUR	MIX AFRICAN/EUROPEAN	9	72	0.125
PUR	NORTH ASIA	3	72	0.041667
PUR	NEAR EAST	1	72	0.013889
TSI	EUROPE	70	83	0.843373
TSI	NEAR EAST	13	83	0.156627
YRI	AFRICA	88	88	1

### Mitochondrial haplogroup determination for the CES data

The Infinium QC array also includes 141 mitochondrial SNP markers. Comparing mitochondrial haplogroups and polymorphisms is an excellent way for determining sample identity and detecting sample mix-ups. Furthermore, mitochondrial haplogroup and polymorphisms are excellent fit for an inference of ethnic origins [23, 24]. We therefore assessed the ability of Infinium QC array to determine the mitochondrial haplogroup. In this analysis, we determined the haplogroups of 33 samples for which we generated both Infinium QC and clinical exome sequencing (CES) data. We ran HaploGrep 2 (http://haplogrep.uibk.ac.at) [25] on the Infinium QC data and compared the respective haplogroup assignments with the haplogroup calls made by Phy-Mer (https://github.com/MEEIBioinformaticsCenter/phy-mer) [26] using the CES data of very high-depth of mitochondrial genome coverage. Phy-Mer haplogroup calls made upon entire mtDNA sequence are accurate by definition, thus, providing for a gold standard. For each of the 33 samples, Phy-Mer determined highly specific haplogroups (Table 6). In contrast, haplogroup calls made by HaploGrep 2 using the 141 SNP markers lack specificity, with accuracies limited to the most general haplogroup branches.

**Table 6:** Haplogroups for 33 in-house samples using 143 markers from the Infinium QC array (HaploGrep 2) and all sequence data (Phy-Mer)

Sample ID	HaploGrep 2 (Human QC array)	Phy-Mer (CES data)
CPM10	C1d1	C1d1c1
CPM11	HV	B4a1a1
CPM12	N	A2w1
CPM13	H	H5a3b
CPM14	N	A2d1
CPM15	N	A2–64
CPM16	N	W1
CPM17	N	A2–64-@153
CPM18	C1b14	C1b14
CPM19	HV2	B4c1b2a2
NA12878	H	H13a1a1a
CPM20	N	A2r
CPM21	D4	D1h1
CPM22	D4	D1
CPM23	C1c	C1c
CPM24	B2	B2v
CPM25	T	T2b
CPM26	C	C1b7a
CPM27	H	H48
CPM28	N	A2
CPM29	M	M7c1a4a
CPM30	HV	R9b2
CPM31	M	M7c1a4a
CPM32	C	C1d
CPM33	C	C1d
CPM34	C	C1d-194
CPM36	N	A2
CPM4	L3	L3b1a
CPM5	D4j	D4j5
CPM6	L2a1c	L2a1c5
CPM7	A5	A5a
CPM8	N	A2w1
CPM9	K	K1a4b1

### Comparison of self-reported ancestry and GPS-derived provenance in CES data

The Children’s Hospital Los Angeles (CHLA) is located in a metropolitan region with very high ethnic diversity. Self-reported ethnicities are frequently wrong, rendering additional challenges for the proper interpretation of candidate variants in our Clinical Exome Sequencing test. An analysis of 24 CHLA samples with self-reported ancestry, which is a-priori assumed to be inaccurate, confirmed regional assignments of samples, with cautionary notes on sample ethnicity. In particular, one African American sample was identified as African (Kenya), one Armenian as Kuwaiti, a Chinese sample was mapped as Japanese, and a Filipino as Vietnamese. Among 15 Hispanic patients of Mexican, mixed or unspecified origin, four were identified as Mexican, nine as Peruvian, one as Indian, and one as Abkhazian. One Caucasian sample was identified as Iberian, and three Indonesian specimens were identified as either Vietnamese (*n* = 2) or Chinese (*n* = 1). The only Korean patient was identified as Chinese. These results again highlighted the potential utility of the Infinium QC array in a molecular diagnostic laboratory.

### Predicted and self-reported gender

In 37 clinical samples that underwent Clinical Exome Sequencing test and had gender data available, the analysis with Infinium QC correctly matched the self-reported gender of all samples except one. The detailed examination of this specimen revealed a clerical error introduced during sample metadata processing, which serves as another great example of Infinium QC utility for detection and correction of errors with potentially deleterious or even disastrous effects on clinical decisions.

### Sample processing errors unearthed using Infinium QC array

In addition to the gender mix-up described above, in preparation of this manuscript, our pipeline was useful in identification of other errors that otherwise would be very difficult to discover. Comparing the Infinium QC data with the CES data set of one of the patients revealed the mismatch and the mix-up. Investigation of sample identities revealed that, in our own data processing system, the same identifier was erroneously assigned to two of the patients, one male and one female. This error was corrected later.

Second, we have identified a misprint in the “Siblings” column in the 1000 Genomes pedigree file (ftp://ftp.1000genomes.ebi.ac.uk/vol1/ftp/technical/working/20130606_sample_info/20130606_g1k.ped). In the last row of the Table 7, NA20336 sibling should actually be NA20334, as verified by concordance analysis and kinship coefficient calculations later.

**Table 7:** 1000 Genomes records showing error in the database

Family ID	Individual ID	Paternal ID	Maternal ID	Gender	Phenotype	Population	Relationship	Siblings	Second Order	Third Order	Other Comments
2484	NA20334	0	0	2	0	ASW	mother	NA20336	NA20337	0	0
2484	NA20335	0	NA20334	1	0	ASW	child	0	NA20336	0	0
2484a	NA20355	0	0	2	0	ASW	unrel	0	0	0	0
2485	NA20336	0	0	2	0	ASW	mother	NA20344	NA20335	0	0

## Discussion

Genotyping is the process of determining the set of gene variants – the genotype – present in individual genomes by examining certain nucleotide positions within the sequence of their DNA. Low-density (LD) genotyping arrays already proven a cost-effective solution for a variety of applications, for example, in whole-genome based prediction of traits in agriculturally important animals and plants [27–30]. In particular, the Illumina BovineLD BeadChip, covering as little as 6909 variants, have found its use in dairy and beef breeds by providing accurate imputation of genotypes previously discerned by higher density arrays. This chip has dramatically lowered the cost of implementing genomic selection in cattle [30].

However, no low-density chip has been yet available for human research. Here we present the results of an evaluation of the performance of the first human LD genotyping array, Illumina’s Infinium QC Array-24 BeadChip (Infinium QC) and its validation as an aid for the quality control (QC) in a variety of experimental and clinical settings. Due to rapidly increasing turnover of processed samples, the cost-efficiency of QC procedures is essential for the standardization and simplification of NGS workflows. In this study, the need for performance evaluations of the Infinium QC arrays was driven primarily by growing demands of a molecular diagnostic laboratory.

Here we focused on ethnicity determination, sample identity, sample-specific variant call accuracy, sample relatedness, and gender determination, with a specific emphasis on ethnicity determination. Accurate determination of ethnicity in the context of genetic diagnosis is of particular importance. Under-appreciation of genetic diversity in the individuals of African ancestry, for example, has led to a significant number of cases of genetic misdiagnosis [31].

Recently, the lack of the knowledge of genetic diversity in different populations or ethnic groups got addressed by the release large, comprehensive reference databases such as ExAC [32], which provides accurate estimates of allele frequencies in a number of ethnic groups or populations. For individual patients, clinical determination of the pathogenicity of a variant critically depends on precision of ethnicity calls. In many cases, self-reported ethnicity labels are not reliable. A combination of Infinium QC with the GPS and reAdmix algorithms for ethnicity determination provided necessary reliability for pathogenicity calling in the Clinical Exome Sequencing Moreover, simultaneously acquired sample-level QC measures allowed us to control for variant call accuracy, potential sample mix-ups, possible gender mix-ups, and sample relatedness.

Notably, here we did not assess the performance of this array in non-QC applications, which rely upon the SNP markers in ADME, blood group, fingerprint, linkage, and extended MHC categories. The utility of the Infinium QC array, therefore, is potentially much wider than the QC. The ADME marker category is especially interesting, as may serve as a basis for subsequent development of cost-effective pharmacogenomics platform.

The Infinium QC array, on the other hand, would clearly benefit from further improvements of its content. In particular, our analysis revealed that the 141 mitochondrial markers on the array are far from being adequate for accurate determination of the mitochondrial haplogroups. Adding a limited number of haplogroup-defining SNPs (http://phylotree.org) may dramatically improve its performance. Additionally, we identified a number of under-performing SNP markers, which are the candidates for replacement.

## Conclusions

In conclusion, systematic evaluation of the performance of the low-density Infinium QC chip, which contains close to 16 K of SNP markers, indicated that low-density chips are suitable cost-effective alternative to high-density arrays for sample level variant calling clinical data QC. Infinium QC chip allows ethnicity determination on a subcontinental scale and is useful for establishing the sample identity as well as for gender and relatedness determination. To increase overall quality of analysis, we recommend removal of a subset of consistently under-performing variants. To expand utility of this low-density chip even further, we recommend an expansion of ADME and mitochondrial haplogroup markers. Inexpensive Infinium-like low-density human chips have a potential to become Swiss army knife type of genotyping assays suitable for many applications, requiring high-throughput assays.

## Additional files

Additional file 1: “List of excluded markers”. Collection of consistently underperforming markers recommended for removal (TXT 4 kb)

Additional file 2: “Additional figures and tables”. This file contains detailed description of Human QC markers, theoretical values of kinship coefficient between related individuals, and details of ethnicity determination analysis using GPS and reAdmix tools, including continent-specific tables and figures (DOCX 2640 kb)

Additional file 3: “Statistic for sample pairs”. Kolmogorov-Smirnov statistic for matched and mismatched sample pairs, the distributions of concordance rates (TXT 7 kb)

## Abbreviations

ASW: Americans of African Ancestry in SW USA
CES: Clinical exome sequencing
CEU: Utah Residents with Northern and Western Ancestry
CHB: Han Chinese in Beijing, China
GIH: Gujarati Indian from Houston, Texas
JPT: Japanese in Tokyo, Japan
MXL: Mexican Ancestry from Los Angeles, USA
NGS: Next generation sequencing
PUR: Puerto Ricans from Puerto Rico
SNP: Single nucleotide polymorphism
TSI: Toscani in Italy
YRI: Yoruba in Ibadan, Nigeria
