# Supplementary material for: Clinical utility of the low-density Infinium QC genotyping Array in a genomics-based diagnostics laboratory
Source: BMC Med Genomics. 2017 Oct 6;10:57. doi: 10.1186/s12920-017-0297-7 (PMC5639583; doi:10.1186/s12920-017-0297-7)
Supplement: Supplementary file 2 — “Additional figures and tables”. This file contains detailed description of Human QC markers, theoretical values of kinship coefficient between related individuals, and details of ethnicity determination analysis using GPS and reAdmix tools, including continent-specific tables and figures (DOCX 2640 kb) [file 12920_2017_297_MOESM2_ESM.docx]

Supplemental materials for

### Clinical Utility of the Low-Density Infinium QC Genotyping Array in a Genomics-based Diagnostics Laboratory

Petr Ponomarenko^1#^, Alex Ryutov^2#^, Dennis T. Maglinte^2,^ Ancha Baranova^3-5^, Tatiana V. Tatarinova^1,5*^, Xiaowu Gai^2,6*^

1. University of La Verne, La Verne, California, USA
2. Center for Personalized Medicine, Department of Pathology and Laboratory Medicine, Children’s Hospital Los Angeles, Los Angeles, California, USA
3. School of Systems Biology, George Mason University, Fairfax Virginia, USA
4. Research Center for Medical Genetics, Moscow, Russia
5. Atlas Biomed Group, Moscow, Russia
6. Department of Pathology and Laboratory Medicine, USC Keck School of Medicine, Los Angeles, California, USA

# joint first authors

*Joint last/corresponding authors ([ttatarinova@laverne.edu](mailto:ttatarinova@laverne.edu); [xgai@chla.usc.edu](mailto:xgai@chla.usc.edu))

# Description of SNP categories

**Pharmacogenomics biomarkers (N = 1,009)** were selected from the PharmADME.org database according to the list of most common requests from Illumina's collaborators.

**Ancestry Informative Markers (AIMs) (N = 2,910)** were comprised from two sources. The first source, "African American vs. European Ancestry", is a grid of 3,388 markers with more or less even distribution on chromosomes, with an approximate density of one per Mb, and the strong ability to differentiate samples of African and European ancestry deposited in the 1000 Genomes Project [1]. Among these, the markers previously included in the Illumina Omni 2.5M array were favored, and the markers represented by A/T or G/C alleles avoided. The second set, capable to sort out Native American versus European Ancestry, contains 1,000 markers selected to be in low linkage disequilibrium to one another (defined as R^2^ ≤ 0.1 in Native American populations) and at least 250 kb apart from each other. SNPs with a significant heterogeneity of the frequencies in same-continent populations were excluded. In this subset, all markers were previously genotyped in three samples of European ancestry and six samples of Native Americans. Among 2,910 AIMs, there was a bias for autosomal locations within coding regions.

**Blood Group Markers (N = 1,659)** were retrieved from the Blood Group Antigen Gene Mutation Database (dbRBC) [2] maintained by NCBI. This set of markers covers 51 genes and is capable to differentiate 34 blood groups including less common ones, such as the Chido/Rodgers Blood Group System and C4 complement.

**Sex chromosomes**. This group includes 1,840 variants located on the X chromosome, 1,401 on Y chromosome and 535 from the pseudoautosomal regions PAR1, PAR2, and PAR3 present in both sex chromosomes.

**Fingerprinting markers (N = 477)**. These “high MAF no LD” variants were submitted by the Population Architecture using Genomics Epidemiology (PAGE) Consortium [http://www.cstl.nist.gov/strbase/SNP.htm](http://www.cstl.nist.gov/strbase/SNP.htm%20) and <http://alfred.med.yale.edu/alfred/index.asp>.

**Linkage markers (N = 5,486)** were taken from a previous Illumina product HumanLinkage and represent common variants most likely to be correctly imputed. <http://support.illumina.com/content/dam/illumina-marketing/documents/products/appnotes/appnote_imputation.pdf>

**Extended set of MHC markers (N = 930).** These markers reside within an extended Major Histocompatibility Complex (MHC) region (8Mb) and are indispensable to determine histocompatibility and predisposition to a variety of chronic diseases.

**Mitochondrial markers (N = 141)**. Maternally inherited variations of a mitochondrial genome constitute a set of distinct signatures known as mitochondrial haplogroups proven extremely valuable in discerning human evolution and migration patterns, and thus used extensively in forensics [3-6].

# Concordance of variant calling between platforms

We compared the Infinium QC data with the 1,000 Genomes WGS, Omni 2.5 (OMNI)and Affymetrix 6.0 (AFFY) microarray data. Venn diagrams of commonly called variants and concordance histograms shown on Figure 1 and Supplementary Figure 1, respectively. The histograms include the concordance for matched and mismatched (simulating accidental sample swap) sample pairs on parent-child, sibling, family, population and other levels of relatedness. Infinium QC microarray is comprised of 15,949 markers covering 15,837 unique loci. Concordance was calculated for chromosomal positions. Call data was combined for positions covered with more than one marker and multi-allelic calls were removed from comparison.

Concordance of genotype calls between Infinium QC and OMNI, AFFY 6.0 and NGS (using 1000 Genomes Project data) (Genomes Project, Auton et al., 2015)was found to be 99.63%, 99.66% and 99.39% correspondingly when only non-missing bi-allelic calls between both sets are compared (except for the Y chromosome comparison between the Infinium QC and 1000 Genomes data, which has a concordance of 95.68%). These concordance values were calculated based on 9,166, 3,290 and 12,820 (only 47 for Y on 1KG) loci that were bi-allelic within and between each pair of datasets correspondingly.

Percentage of genotype calls missing in one or both datasets in each pair of Infinium QC data vs OMNI, AFFY and 1KG is 6.44%, 0.56% and 0.13% correspondingly (3.56% for Y chromosome from 1KG). There were less than 1% discordant calls, and most of them were possibly reported on the wrong strand (e.g., A/A instead of T/T). These loci can be found in Supplementary Tables 1 and 2.

Supplemental Table 4 shows positions with highest number of mismatches between Infinium QC and 1000 Gеnomes. For same positions, number of mismatches of Infinium QC vs Affymetrix data is also shown for comparison. All except chrX:120474720* (rs6649211) were frequently mismatched when Infinium QC is compared to 1000 Genomes and Affymetrix. Assuming 1000 Genomes genotype calls represent the gold standard, these positions, their markers and probes may require comprehensive analysis in Infinium QC or better yet, should be removed from concordance analysis.

We observed a strange incidence of discordant heterozygous calls on the Y chromosome. Focusing only on the regions that are covered by the whole-exome sequencing (WES) increases the concordance above the 99% range for the Y chromosome. WES filtering for other chromosomes does affect the concordance between platforms inconsistently, making it higher for comparison with AFFY 6.0 and Y chromosome calls in 1000 Genomes data, while decreasing concordance between Infinium QC vs. OMNI and 1,000 genomes data (excluding the Y chromosome). Outside of the WES regions on the Y chromosome all discordant calls were heterozygous in Infinium QC with one of the alleles identified correctly. Illumina states that genotype calls for female samples on Y chromosome will be performed and result in low quality calls, and thus should be removed. This can be used to confirm gender of the sample.

**Infinium QC vs AFFY 6.0** concordance is 99.66% on the 2,526 marker positions that are shared between them and bi-allelic internally and between them on non-missing calls (there are 1,637,639 calls that are non-missing in both datasets based on 652 individuals present in both data sets). 9,313 calls are missing in one or both sets, this is 0.56% of all calls. Out of 5,607 non-missing mismatching genotypes, 1,789 are matching by one of the alleles (31.9%). Filtering by the WES regions results in higher concordance of 99.9104% for the subset of markers (132,677 genotype calls are matching out of 133,660 in total and 132,796 non-missing). After the WES filtering, out of 119 non-missing mismatching genotypes 116 are matching by one of the alleles (97.5%).

**Infinium QC vs. OMNI** concordance is 99.63% on 7,781 marker positions (4,806,200 non-missing calls in both sets out of 5,112,117 from 657 individuals). Missing calls in one or both 5.98% or 305,917 marker positions. Out of 17,782 non-missing mismatching genotypes, 2,061 are matching by one of the alleles (11.5%). After filtering for the WES regions, we get 465,156 overlapping calls, 435,185 non-missing in both file sets, 423,801 concordant, for a concordance rate of 97.38%. Out of 11,384 non-missing mismatching genotypes after the WES filtering, 96 are matching by one of the alleles (0.8%).

**Infinium QC vs. 1,000 Genomes.** There are 503 individuals present in the latest release of the 1,000 Genomes dataset (combined NGS and genotyping data) and genotyped with the Infinium QC as well. concordance is 92.32% on 12,820 overlapping marker positions on all chromosomes except Y (based on 5,071,268 non-missing calls from both sets of 503 individuals). 7,020 genotypes, or 0.13%, are missing in one or both. Out of 30,829 non-missing mismatching genotypes 12,901 are matching by one of the alleles (41.8%). After the filtering for WES, the quality is lower – 98.63% (592,031 overlapping calls, 591,461 non-missing in both file sets, with 583,345 concordant calls). The size of the WES subset contains only 11.6% of the original number of markers. After the WES filtering, out of 8,116 non-missing mismatching genotypes 1,623 are matching by one of the alleles (20%). Therefore, there is no relationship between WES filtering and percentage of calls where only one allele was correctly identified.

On the Y chromosome, the concordance with 1000 Genomes is only 95.68% based on 11,458 non-missing marker positions from 252 individuals. 386 calls (3.26%) are missing. On the Y chromosome, we observe in Infinium QC – 484 out of 485 mismatching genotypes are heterozygous in Infinium QC and in all of them one of the alleles was correctly identified. Such calls have low quality scores.

After filtering for the Y-chromosomal exonic regions, we get only 756 overlapping calls with 732 non-missing in both file sets. 731 of them are concordant for a concordance rate of 99.86%. The only discordant call was made when both are not missing T/C in Infinium QC data while T/T in 1KG data. Therefore, we observed that exonic regions of the Y chromosome have much higher concordance compared to intergenic and intronic regions, but the number of markers in exonic regions is too small to conduct statistical tests. Genotype call quality for discordant SNPs is higher than average (0.776). Only 4 out of 24 highly discordant SNPs between Infinium QC and 1KG data that were also present on the OMNI chip had genotype scores below average. VCF files created by different software may contain reversed genotypes, e.g., genotype may be reported as a minor/major allele or as a base/alt allele. Therefore, calls with reversed genotypes were counted as concordant. The procedure to flip genotypes to account for Illumina's top/bottom designation was performed specifically for ancestry determination to normalize data with Genographic chip; it was not needed for concordance calculation. We prepared a list of markers that were underperforming in concordance between different platforms. This included markers of highly polymorphic regions, pseudoautosomal regions, as well as markers with probes mapped to multiple positions according to Illumina specifications. This list is shown in the Supplemental Table 4. These loci are located in repetitive or highly polymorphic regions like MHC Class I cluster of genes (specifically HLA- A, B and C that are most polymorphic in MHC I genes) and GPCR class A cluster of genes.

Suppl. Table 1: Overlapping markers between Human QC array and other experimental platforms and public datasets.

| Platform Comparison | Number of Common Variants Between Platforms | Number of samples | Number of Variants in the Comparison Platform Only |
| --- | --- | --- | --- |
| Infinium QC vs. Affymetrix | 3290 | 652 | 33 |
| Infinium QC vs. Omni (Illumina) | 9166 | 657 | 159 |
| Infinium QC vs 1000 Genomes | 12820 | 503 | 908 |
| CPM Infinium QC vs CPM CES | 761 | 33 | 114862 |

**Population-wise sample concordance**

When pairwise concordance values were calculated for a large set of samples, a bi-modal distribution was observed for mismatched sample pairs (Supplemental Figure 1). This can be explained by considering the pairwise concordance between different populations. The 1000 Genomes pedigree file contains population data for each sample; these populations are combined into five super-populations – African, Admixed American, East Asian, European and South Asian. A procedure similar to the family concordance calculation was applied to the populations. Sample pairs belonging to the same super-population were extracted from the set of mismatched pairs, and analyzed with the population-specific histograms. The population concordance histograms for the Infinium QC vs 1000 Genomes comparison are presented in the Supplemental Figure 1.


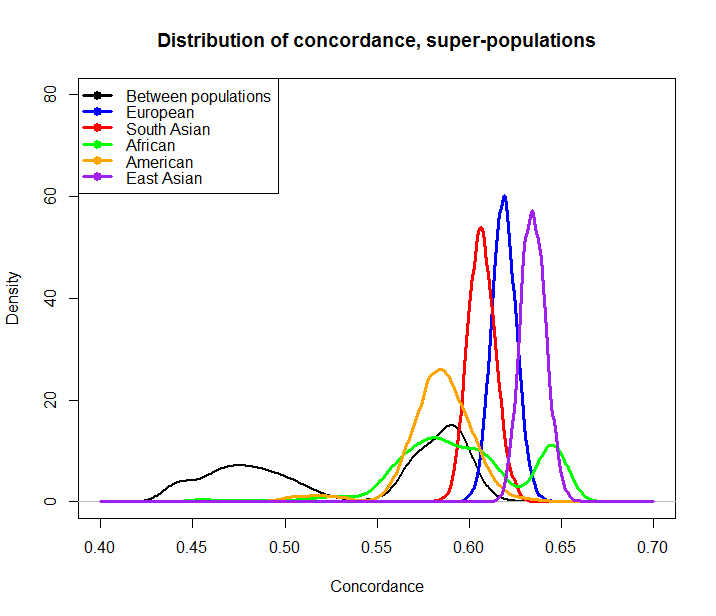


Suppl. Figure 1: Concordance between every possible pair of samples within populations, self-hits excluded

As can be seen in Supplemental Figure 1, the population-wise concordance values are tightly clustered, especially for the Eastern Asian, European and South Asian super-populations. Sample pairs belonging to different populations were separated into ten possible groups of mismatched super population pairs. The average concordance values for the mismatched pairs are shown in Supplemental Table 2.

Suppl. Table 2: Population-wide concordance. AFR: African American; AMR: Admixed American; EAS: East Asian; EUR: European; SAS: South Asian

|  | AFR | AMR | EAS | EUR | SAS |
| --- | --- | --- | --- | --- | --- |
| AFR | 0.559928 | 0.417711 | 0.431412 | 0.390875 | 0.417188 |
| AMR |  | 0.55099 | 0.543173 | 0.558732 | 0.550021 |
| EAS |  |  | 0.60714 | 0.539716 | 0.555402 |
| EUR |  |  |  | 0.597023 | 0.567043 |
| SAS |  |  |  |  | 0.576606 |

Clearly, the following comparisons resulted in different concordance averages:

1. Concordance of Africans vs. “all other super populations” is lower (0.39-0.43)

2. Concordance values inside same population are higher (0.55-0.61)

3. Other pairings are similar in concordance with within-population concordance (0.54-0.57)

Standard deviation of concordance distribution between different samples inside African population is much greater than for other populations. These groups of concordance were responsible for the two modes of the unrelated sample pairs concordance distribution.

Suppl. Table 3: Comparison of Infinium QC data with OMNI, AFFY 6.0, and 1,000 Genomes (phase 3) on HapMap samples

| Comparison with Infinium QC | OMNI | AFFY 6.0 (WES) | 1KG no Y | 1KG only Y |
| --- | --- | --- | --- | --- |
| Concordance, % | 99.63% (97.38%) | 99.66% (99.91%) | 99.39% (98.63%) | 95.68% (99.86%) |
| shared markers | 7,781 (708) | 2,526 (205) | 10,096 (1,177) | 47 (3) |
| non-missing genotype calls | 4,806,200 (465,156) | 1,637,639 (132,796) | 5,071,268 (591,461) | 11,458 (732) |
| matching samples | 657 | 652 | 503 | 252 |
| missing genotype calls | 305,917 (29,971) | 9,313 (864) | 7,020 (570) | 386 (24) |
| missing genotype calls, % | 5.98% (6.44%) | 0.56% (0.64%) | 0.13% (0.09%) | 3.26% |
| non-missing mismatches | 17,782 (11,384) | 5,607 (119) | 30,829 (8,116) | 485 (1) |
| only one allele matches | 2,061 (96) | 1,789 (116) | 12,901 (1,623) | (1) |
| % one allele matches out of all genotype mismatches | 11.5% (0.8%) | 31.9% (97.5%) | 41.8% (20%) | (100%) |

Suppl. Table 4: Infinium QC vs. 1000 Genomes (1KG), Affymetrix and OMNI results, thirty most discordant positions. Based on 503 samples in the intersection of Infinium QC and 1000 Genomes dataset, as well as 652 samples for Affymetrix and 657 with OMNI. Average genotype call score across Infinium QC chip is 0.78 with standard deviation of 0.21, minimum of 0.1 and maximum of 0.98. Empty cells correspond to positions not covered by OMNI or Affymetrix datasets.

| Chromosome: Position | Marker name | Most discordant genotype calls between Infinium QC and | | | Average genotype call score | |
| --- | --- | --- | --- | --- | --- | --- |
|  |  | 1KG | OMNI | AFFY |  |  |
| chr4:69512637 | rs4148271 | 451 | 596 |  | 0.915051523 |  |
| chr7:87133470 | rs17064 | 442 | 560 |  | 0.92561961 |  |
| chr6:29712759* | exm-rs2844845 | 395 | 48 | 520 | 0.959497865 |  |
| chr19:41354533 | rs1801272 | 489 |  |  | 0.662827184 |  |
| chr8:145639681 | rs1871534 | 464 |  |  | 0.871873314 |  |
| chr6:32411846 | exm-rs2239802 | 352 | 437 |  | 0.83848067 |  |
| chr13:20901724* | rs1335873 | 323 | 34 | 425 | 0.849843877 |  |
| chr15:74710485 | rs2072649 | 410 |  |  | 0.949527 |  |
| chr3:102017934 | rs1479371 | 326 |  | 406 | 0.897622045 |  |
| chr6:32485705 | JHU_6.32485704 | 398 |  |  | 0.614315374 |  |
| chr11:5099393 | rs10500617 | 308 | 397 |  | 0.940991341 |  |
| chr3:151899704 | rs161792 | 293 |  | 388 | 0.94297633 |  |
| chr6:32609698 | JHU_6.32609697 | 384 |  |  | 0.515204873 |  |
| chr1:25745061 | 1:25745061-T-C | 375 |  |  | 0.281301283 |  |
| chr13:103528002 | rs17655 | 295 | 368 |  | 0.903758359 |  |
| chr17:43131480 | rs4793172 | 284 |  | 368 | 0.94741824 |  |
| chrX:2707978 | rs311166 | 367 |  |  | 0.200194158 |  |
| chr19:48374551 | rs296365 | 278 | 361 |  | 0.913563243 |  |
| chr19:582927 | rs8259 | 281 | 358 |  | 0.960199669 |  |
| chr13:106938411* | rs354439 | 262 | 34 | 352 | 0.934711838 |  |
| chr1:25720045 | 1:25720045-G-C | 352 |  |  | 0.316731322 |  |
| chrX:86086809 | kgp22826616 | 351 |  |  | 0.513480317 |  |
| chr5:101671415 | rs1584717 | 272 |  | 351 | 0.887001731 |  |
| chr3:961782 | rs1357617 | 342 |  |  | 0.88096531 |  |
| chr2:101010082 | rs3748930 | 266 | 340 |  | 0.933418134 |  |
| chr11:124115370 | rs2512276 | 262 |  | 338 | 0.942959009 |  |
| chr6:32629371 | JHU_6.32629370 | 337 |  |  | 0.600026299 |  |
| chr2:215645464 | rs2229571 | 264 | 335 |  | 0.953172714 |  |
| chr11:115207176* | rs10488710 | 251 | 26 | 325 | 0.865332735 |  |
| chr4:190318080* | rs1979255 | 251 | 30 | 324 | 0.832853611 |  |

Most discordant calls are consistent across pairs of different datasets (see Supplemental Tables 1 and 4). Only four out of top 30 most discordant Infinium QC vs 1000 Genomes markers are found both in OMNI and Affymetrix datasets. It is very interesting that for these four markers OMNI and Affymetrix also discordant between each other. This demonstrates the repetitive and polymorphic nature of their origin. Variants were excluded based on five conditions: discordant more than 10% when Infinium QC compared with 1000 Genomes dataset, mutations in HLA gene regions that are highly paralogous pseudoautosomal regions, mitochondrial genotype calls with more than one allele called and markers that can be mapped to more than one location in GRCh37 that were not removed because of other reasons

**Kinship calculation**

Suppl. Table 5: Theoretical kinship coefficient

| **Degree of**  **relationship** | **Relationship** | **Coefficient of**  **relationship (r)** | **Kinship**  **coefficient** |
| --- | --- | --- | --- |
| 0 | identical twins; clones | 100% | 0.5 |
| 1 | parent-offspring | 50% | 0.25 |
| 2 | full siblings | 50% | 0.25 |
| 2 | 3/4 siblings or sibling-cousins | 37.50% | 0.1875 |
| 2 | grandparent-grandchild | 25% | 0.125 |
| 2 | half siblings | 25% | 0.125 |
| 3 | aunt/uncle-nephew/niece | 25% | 0.125 |
| 4 | double first cousins | 25% | 0.125 |
| 3 | great grandparent-great grandchild | 12.50% | 0.0625 |
| 4 | first cousins | 12.50% | 0.0625 |
| 6 | quadruple second cousins | 12.50% | 0.0625 |
| 6 | triple second cousins | 9.38% | 0.0469 |
| 4 | half-first cousins | 6.25% | 0.03125 |
| 5 | first cousins once removed | 6.25% | 0.03125 |
| 6 | double second cousins | 6.25% | 0.03125 |
| 6 | second cousins | 3.13% | 0.01565 |
| 8 | third cousins | 0.78% | 0.0039 |
| 10 | fourth cousins | 0.2% | 0.001 |

**Excluded markers**

The list of excluded markers is in the Supplementary file 2.

**Ethnicity prediction**

Supplemental Figures 5-10 and Supplemental Tables 1-2 show the accuracy of GPS prediction of ancestry based on Human QC data. Plots are generated using the Plotly Online Chart Maker, available at [plot.ly](file:///C:\Users\tatia\Dropbox%20(Personal)\HumanQC\REVISION\R2\plot.ly).


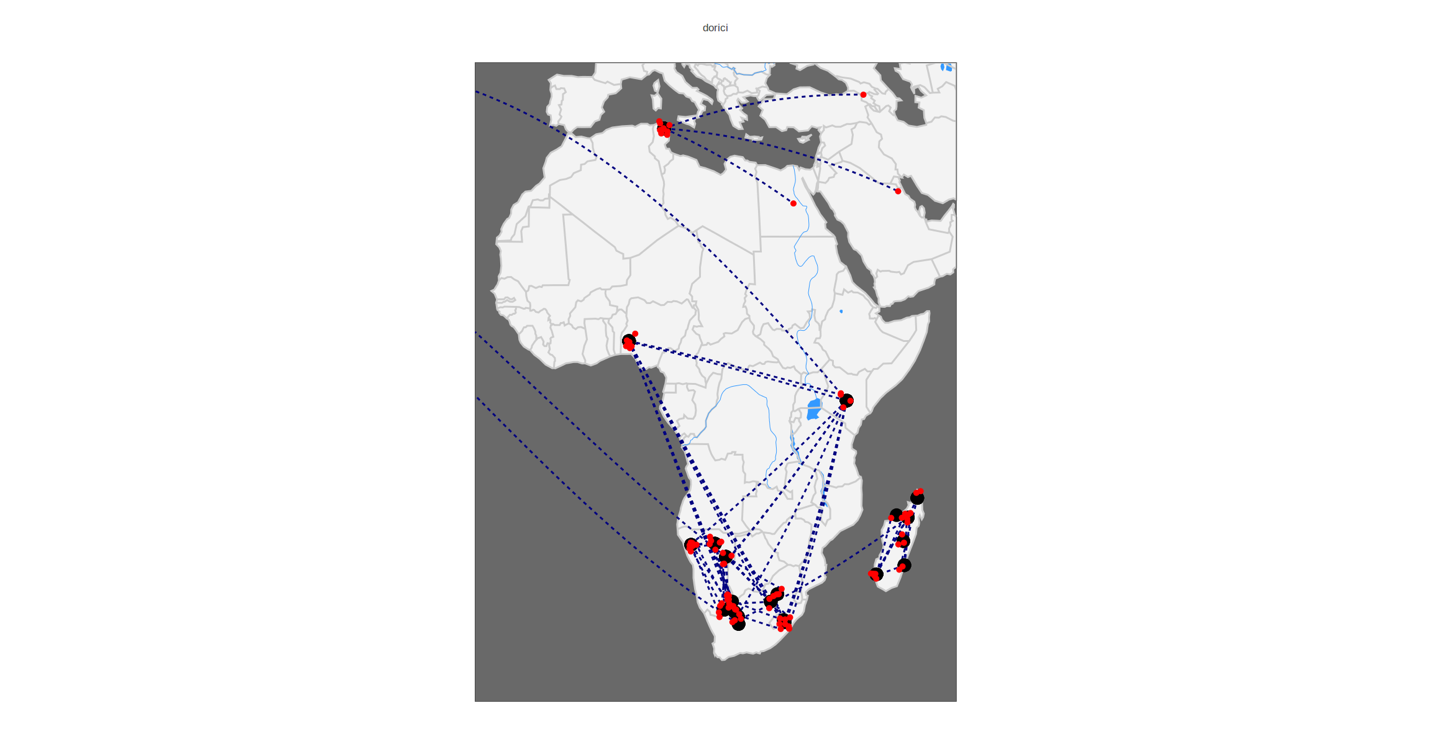


Suppl. Figure 2: GPS accuracy for Africa. Out of 111 African samples the following are mapped outside of the region: African American (2), Bermudian (1), Ingush (1), Puerto Rico (1), and Kuwaiti (4).


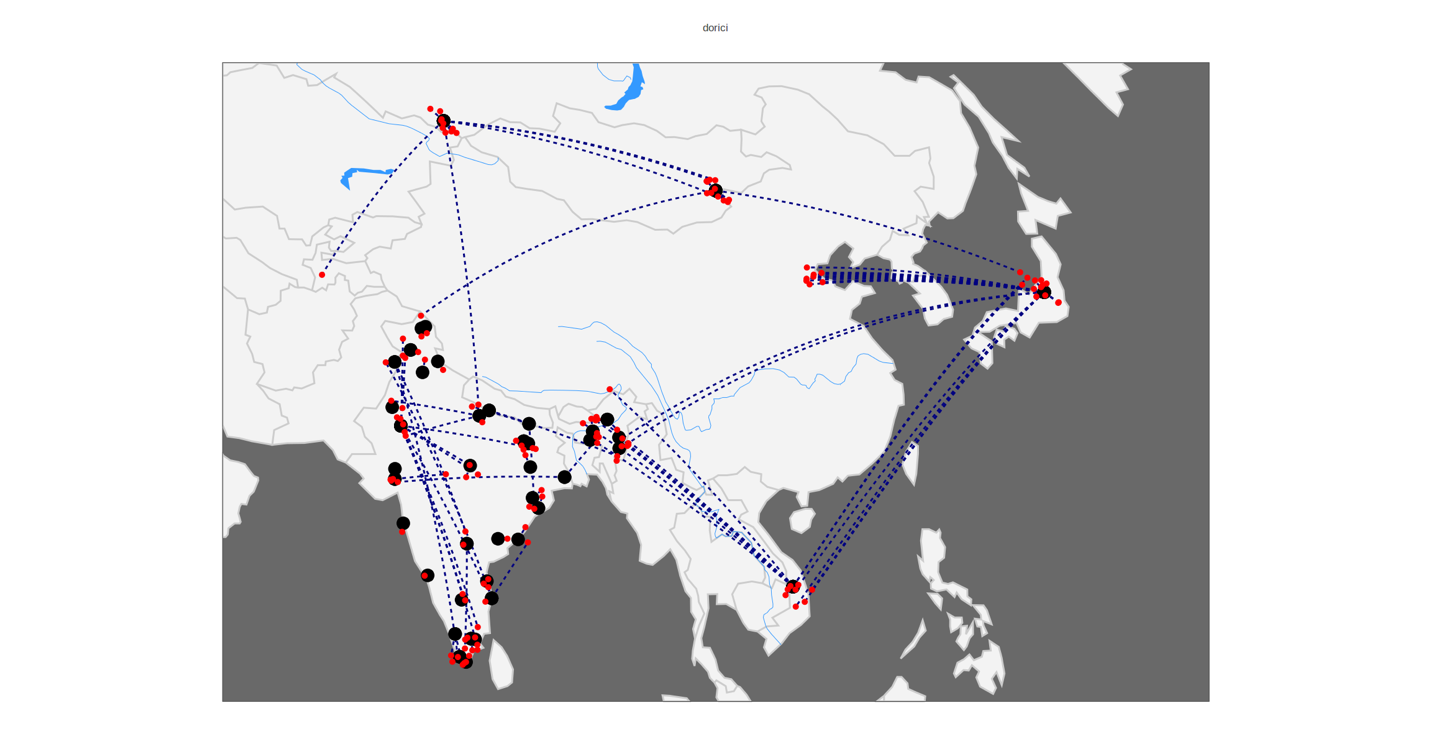


Suppl. Figure 3: GPS accuracy for Asian and Indian samples


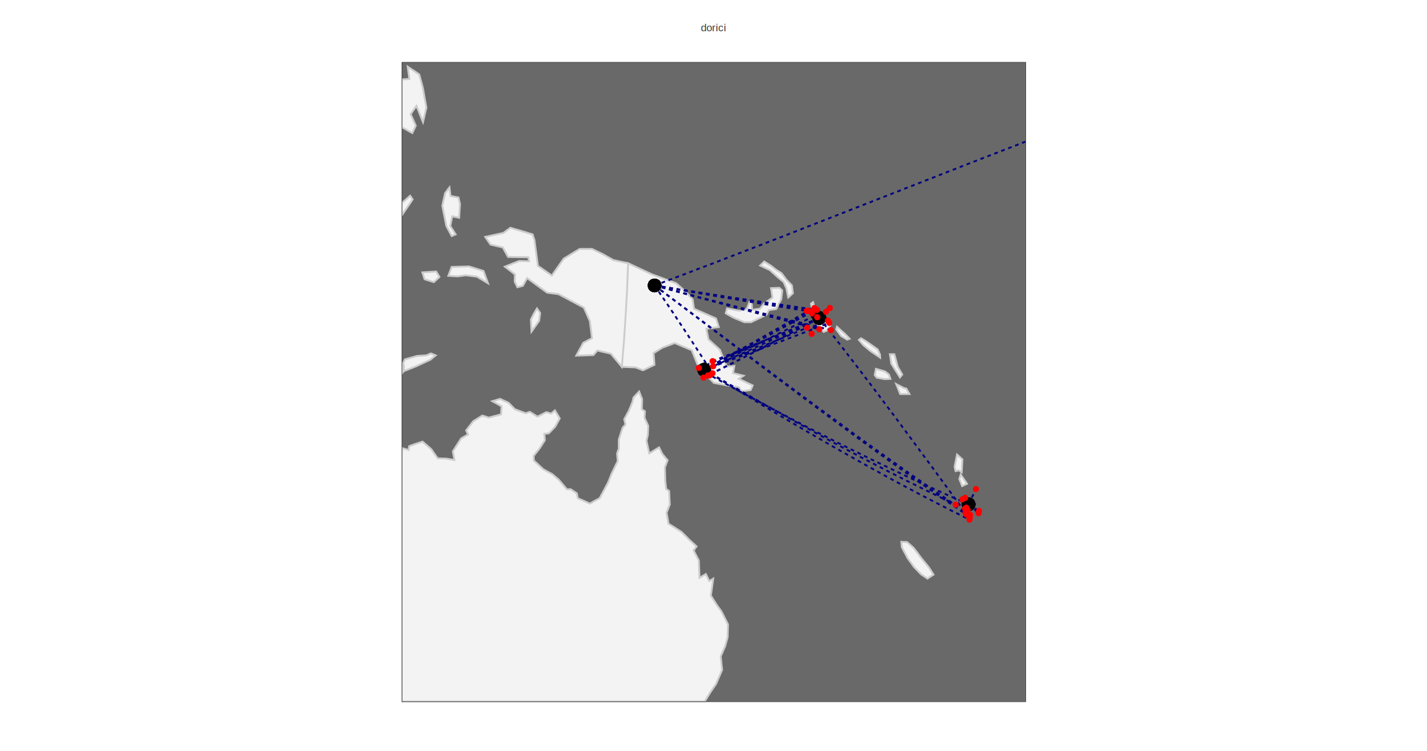


Suppl. Figure 4: Accuracy for Oceania. One sample mapped to Mexico


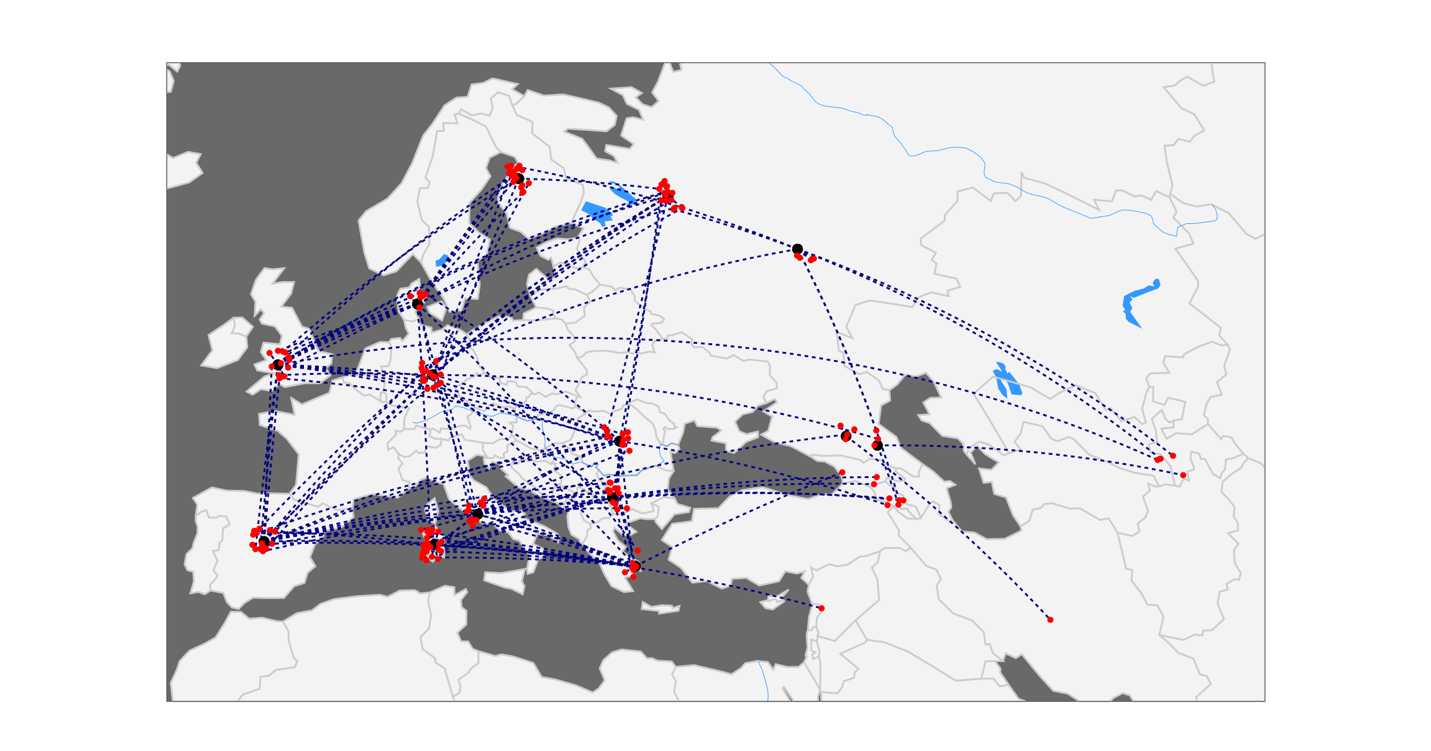


Suppl. Figure 5: Accuracy for Europe


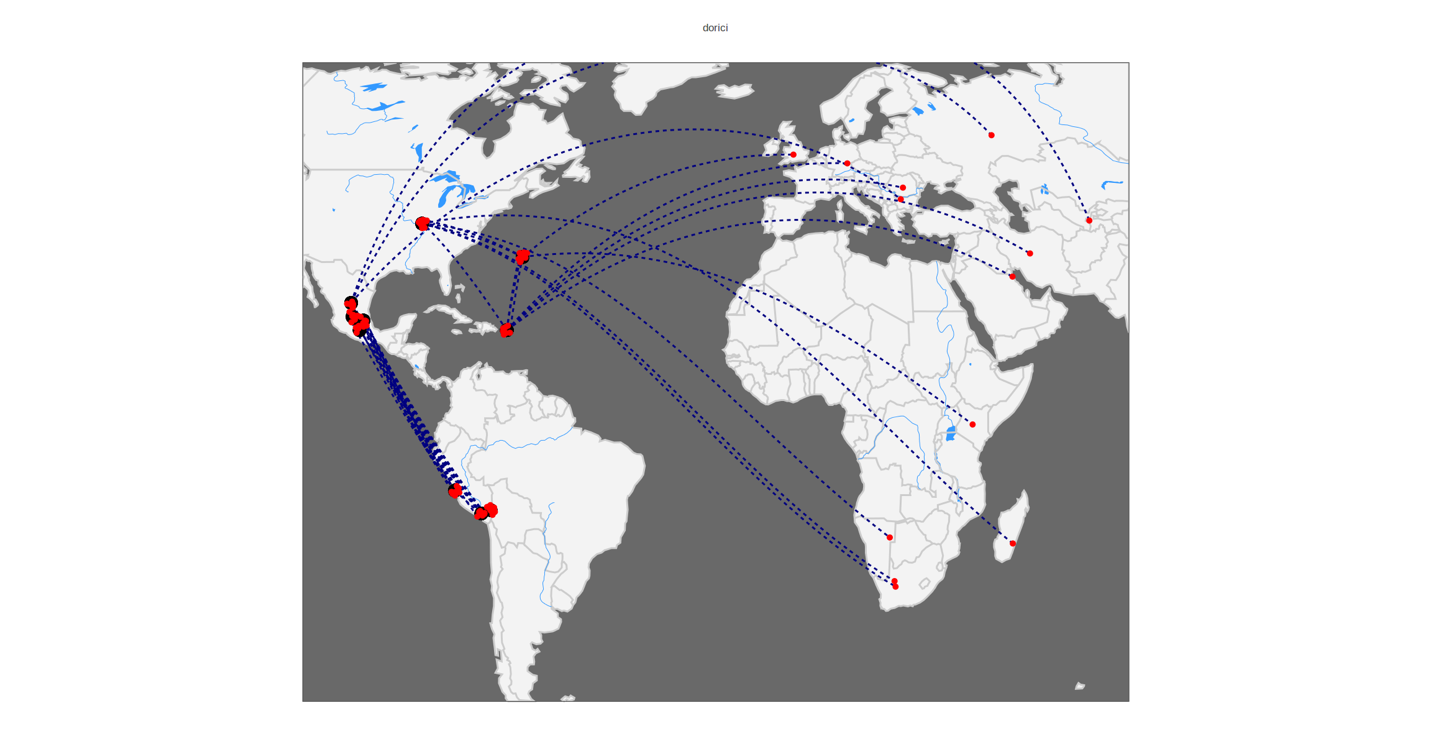


Suppl. Figure 6: Accuracy for America. Native American samples are frequently mis-annotated between Mexican and Peruvian. African American samples can be labeled as European or African, reflecting their ancestry

Suppl. Table 6: Population analysis. Comparison of GPS-predicted ancestry with the reported ancestry.

| REGION | POPUPLATION | %REGION | %POP |
| --- | --- | --- | --- |
| AFRICA | Ambilobe Madagascar | 100% | 50% |
|  | Andilambe Madagascar | 100% | 33% |
|  | Andriesvale Northern Cape Rsa | 67% | 0% |
|  | Antananarivo Madagascar | 100% | 100% |
|  | Askham Northern Cape Rsa | 100% | 100% |
|  | Hereroland Namibia | 80% | 0% |
|  | Johannesburg Gauteng Rsa | 100% | 100% |
|  | Kaokoveld Namibia | 100% | 40% |
|  | Kenya | 92% | 25% |
|  | Kroonstad Free State Rsa | 100% | 0% |
|  | Mahajanga Madagascar | 100% | 100% |
|  | Manakara Madagascar | 100% | 25% |
|  | Reichenau Mission Underberg Kzn Rsa | 100% | 100% |
|  | Schmidtsdrift Northern Cape Rsa | 100% | 25% |
|  | Southeastern Namibia | 100% | 40% |
|  | Toliara Madagascar | 100% | 50% |
|  | Tsumkwe Namibia | 100% | 40% |
|  | Tunisia | 75% | 75% |
|  | Underberg KZN RSA | 100% | 0% |
|  | Witdraai Northern Cape Rsa | 100% | 100% |
|  | Yoruba | 100% | 42% |
| AMERICA | African American | 67% | 67% |
|  | Aymara Uro | 100% | 100% |
|  | Bermudian | 88% | 63% |
|  | Guanajuato Mexico | 100% | 67% |
|  | Egypt | 60% | 60% |
|  | Hidalgo Mexico | 100% | 38% |
|  | Mexican | 75% | 58% |
|  | Morelos Mexico | 100% | 100% |
|  | Peru | 100% | 50% |
|  | Puerto Rico | 73% | 53% |
|  | Quechua | 100% | 9% |
|  | Queretaro Mexico | 100% | 100% |
| ASIA | Altaians | 87% | 67% |
|  | Chinese | 83% | 75% |
|  | Japan | 92% | 42% |
|  | Mongols | 91% | 82% |
|  | Vietnamese | 50% | 33% |
| Europe | Abkhazians | 100% | 50% |
|  | Adi | 100% | 100% |
|  | Bulgaria | 100% | 7% |
|  | Chechens | 100% | 50% |
|  | Dane | 100% | 13% |
|  | Finnish | 100% | 75% |
|  | Georgians | 100% | 75% |
|  | German | 100% | 27% |
|  | Greek | 100% | 13% |
|  | Iberian | 100% | 17% |
|  | Ingush | 100% | 50% |
|  | Iran | 81% | 38% |
|  | Italian | 100% | 20% |
|  | Kuwait | 83% | 11% |
|  | Lebanon | 100% | 27% |
|  | Romania | 100% | 13% |
|  | Russians | 100% | 50% |
|  | Sardinian | 100% | 80% |
|  | Tajikistan | 100% | 69% |
|  | Tatar (Russia) | 93% | 47% |
|  | Uk | 100% | 15% |
| INDIA | Apatani | 100% | 100% |
|  | Binjhal | 100% | 0% |
|  | Bondo | 100% | 100% |
|  | Boro Kachori | 100% | 100% |
|  | Brahmin Bhumihar | 100% | 100% |
|  | Brahmin Goudsaraswath | 100% | 100% |
|  | Brahmin Kanyakubj | 100% | 100% |
|  | Brahmin Mythil | 100% | 0% |
|  | Brahmin Saraswath | 100% | 100% |
|  | Brahmin Saryupareen | 100% | 0% |
|  | Brahmin Utkalya | 100% | 100% |
|  | Brahmin Vadama | 100% | 100% |
|  | Brokpa Bhuddist | 100% | 100% |
|  | Changspa | 100% | 100% |
|  | Charan | 100% | 100% |
|  | Ezhava | 100% | 100% |
|  | Gaddi Rajput | 100% | 100% |
|  | Gond Ori | 100% | 67% |
|  | Gujarati | 100% | 8% |
|  | Halba | 100% | 50% |
|  | Ho | 100% | 100% |
|  | Iyengar Kar | 100% | 50% |
|  | Kalita | 100% | 100% |
|  | Kapu | 100% | 50% |
|  | Karbi | 100% | 100% |
|  | Katkari | 100% | 100% |
|  | Kattunaickan | 100% | 100% |
|  | Khasi | 100% | 100% |
|  | Koya | 100% | 100% |
|  | Kuki | 100% | 100% |
|  | Langia Soura | 100% | 100% |
|  | Majhbi | 100% | 0% |
|  | Meitei | 100% | 100% |
|  | Mogaveera | 100% | 100% |
|  | Munda | 100% | 100% |
|  | Nadar Cape | 100% | 100% |
|  | Naga | 100% | 100% |
|  | Nattukottai Chettiar | 100% | 100% |
|  | Parayar | 100% | 100% |
|  | Parsee | 100% | 100% |
|  | Piraimalai Kallar | 100% | 50% |
|  | Pulayar | 100% | 0% |
|  | Rajput Kinnura | 100% | 100% |
|  | Rajput Rajasthan | 100% | 100% |
|  | Relli | 100% | 100% |
|  | Sattibalija | 100% | 100% |
|  | Tharu | 100% | 50% |
|  | Valayar | 100% | 100% |
| OCEANIA | Nasioi Bougainville | 100% | 40% |
|  | Papua New Guinea Coastal | 100% | 0% |
|  | Papua New Guinea Highland | 89% | 0% |
|  | Vanuatu | 100% | 90% |

Suppl. Table 7: Population analysis

| ORI | PRED_POP | HIT | TOTAL | PERCENT | Region | Continent |
| --- | --- | --- | --- | --- | --- | --- |
| ASW | Bermudian | 47 | 90 | 52% | North America | America |
|  | Kenya | 27 | 90 | 30% | Kenya | Africa |
|  | Puerto Rico | 5 | 90 | 6% | North America | America |
|  | Mandenka | 4 | 90 | 4% | West Africa | Africa |
|  | Yoruba | 4 | 90 | 4% | West Africa | Africa |
|  | Bantu S.E. Zulu | 1 | 90 | 1% | South Africa | Africa |
|  | Ethiopians O | 1 | 90 | 1% | Eastern Africa | Africa |
|  | East Greenlanders | 1 | 90 | 1% | Northern Europe | Europe |
| CEU | German | 14 | 88 | 16% | Western Europe | Europe |
|  | Iberian | 11 | 88 | 13% | Western Europe | Europe |
|  | Orcadian | 8 | 88 | 9% | Northern Europe | Europe |
|  | Bulgaria | 7 | 88 | 8% | Eastern Europe | Europe |
|  | French | 6 | 88 | 7% | Western Europe | Europe |
|  | Lithuanians | 6 | 88 | 7% | Northern Europe | Europe |
|  | Russian | 6 | 88 | 7% | Eastern Europe | Europe |
|  | UK | 5 | 88 | 6% | Northern Europe | Europe |
|  | Romanian | 5 | 88 | 6% | Eastern Europe | Europe |
|  | Finnish | 5 | 88 | 6% | Northern Europe | Europe |
|  | Dane | 5 | 88 | 6% | Northern Europe | Europe |
|  | French Basque | 3 | 88 | 3% | Western Europe | Europe |
|  | Koryak | 2 | 88 | 2% | Far East | Asia |
|  | Spaniard | 1 | 88 | 1% | Western Europe | Europe |
|  | Hungarians | 1 | 88 | 1% | Eastern Europe | Europe |
|  | Tuscan | 1 | 88 | 1% | Western Europe | Europe |
|  | Belorussian | 1 | 88 | 1% | Eastern Europe | Europe |
|  | Hidalgo Mexico | 1 | 88 | 1% | North America | America |
| GIH | Gujarati | 45 | 77 | 58% | India | Asia |
|  | India Meena | 4 | 77 | 5% | India | Asia |
|  | Iyengar Kar | 4 | 77 | 5% | India | Asia |
|  | Indian Velama | 3 | 77 | 4% | India | Asia |
|  | Sindhi | 3 | 77 | 4% | India | Asia |
|  | Majhbi | 3 | 77 | 4% | India | Asia |
|  | India Meghawal | 3 | 77 | 4% | India | Asia |
|  | Kapu | 2 | 77 | 3% | India | Asia |
|  | Valayar | 2 | 77 | 3% | India | Asia |
|  | India Tamil Nadu, Brahmins | 2 | 77 | 3% | India | Asia |
|  | Balochi | 2 | 77 | 3% | Pakistan | Asia |
|  | Burusho | 1 | 77 | 1% | Northern Pakistan | Asia |
|  | India Uttar Pradesh, Muslim | 1 | 77 | 1% | India | Asia |
|  | Pathan | 1 | 77 | 1% | India | Asia |
|  | India Uttar Pradesh, Brahmins | 1 | 77 | 1% | India | Asia |
| MXL | Peru | 53 | 82 | 65% | South America | America |
|  | Hidalgo Mexico | 15 | 82 | 18% | North America | America |
|  | Puerto Rico | 8 | 82 | 10% | North America | America |
|  | Sardinian | 2 | 82 | 2% | Mediterranean | Europe |
|  | Canary Islands | 2 | 82 | 2% | Mediterranean | Africa |
|  | Quechua | 2 | 82 | 2% | South America | America |
| PUR | Puerto Rico | 31 | 72 | 43% | North America | America |
|  | Abkhazians | 1 | 72 | 1% | Caucasus | Europe |
|  | Bermudian | 5 | 72 | 7% | North America | America |
|  | Bulgaria | 1 | 72 | 1% | Eastern Europe | Europe |
|  | Bulgarian | 3 | 72 | 4% | Eastern Europe | Europe |
|  | Canary Islands | 5 | 72 | 7% | Mediterranean | Africa |
|  | Catalonia | 1 | 72 | 1% | Western Europe | Europe |
|  | Cypriots | 1 | 72 | 1% | Mediterranean | Europe |
|  | Egyptians | 2 | 72 | 3% | Northern Africa | Africa |
|  | Greek | 2 | 72 | 3% | Mediterranean | Europe |
|  | Lebanese Akkar Muslim | 1 | 72 | 1% | Near East | Asia |
|  | Lebanese Bent Jbeil Christian | 1 | 72 | 1% | Near East | Asia |
|  | Lebanese Hasbaya Muslim | 2 | 72 | 3% | Near East | Asia |
|  | Lebanese Tyre Christian | 1 | 72 | 1% | Near East | Asia |
|  | Mozabite (Algeria) | 2 | 72 | 3% | North Africa | Africa |
|  | Peru | 1 | 72 | 1% | South America | America |
|  | Russian | 1 | 72 | 1% | Eastern Europe | Europe |
|  | Sardinian | 3 | 72 | 4% | Mediterranean | Europe |
|  | Tunisia | 6 | 72 | 8% | North Africa | Africa |
|  | Ukrainians | 2 | 72 | 3% | Eastern Europe | Europe |
| TSI | Sardinian | 21 | 83 | 25% | Mediterranean | Europe |
|  | Italian | 12 | 83 | 14% | Mediterranean | Europe |
|  | Lebanon | 10 | 83 | 12% | Near East | Asia |
|  | Cypriots | 9 | 83 | 11% | Mediterranean | Europe |
|  | Greek | 6 | 83 | 7% | Mediterranean | Europe |
|  | Tuscan | 5 | 83 | 6% | Western Europe | Europe |
|  | Bulgaria | 5 | 83 | 6% | Eastern Europe | Europe |
|  | Romanian | 2 | 83 | 2% | Eastern Europe | Europe |
|  | Iberian | 2 | 83 | 2% | Western Europe | Europe |
|  | Chechens | 2 | 83 | 2% | Caucasus | Europe |
|  | Abkhazians | 2 | 83 | 2% | Caucasus | Europe |
|  | Ingush | 1 | 83 | 1% | Caucasus | Europe |
|  | Hungarians | 1 | 83 | 1% | Eastern Europe | Europe |
|  | Lithuanians | 1 | 83 | 1% | Northern Europe | Europe |
|  | Lebanese Hasbaya Druze | 1 | 83 | 1% | Near East | Asia |
|  | Kuwait | 1 | 83 | 1% | Near East | Asia |
|  | Catalonia | 1 | 83 | 1% | Western Europe | Europe |
|  | French | 1 | 83 | 1% | Western Europe | Europe |
| YRI | Yoruba | 83 | 88 | 94% | West Africa | Africa |
|  | Kaokoveld Namibia | 5 | 88 | 6% | South-West Africa | Africa |
| CHB | Chinese | 19 | 38 | 50% | Far East | Asia |
|  | Japanese | 17 | 38 | 45% | Far East | Asia |
|  | Miaozu (China) | 2 | 38 | 5% | Far East | Asia |
| JPT | Japanese | 33 | 45 | 73% | Far East | Asia |
|  | Chinese | 12 | 45 | 26% | Far East | Asia |

**References**

1. Genomes Project C, Auton A, Brooks LD, Durbin RM, Garrison EP, Kang HM, Korbel JO, Marchini JL, McCarthy S, McVean GA *et al*: **A global reference for human genetic variation**. *Nature* 2015, **526**(7571):68-74.

2. Patnaik SK, Helmberg W, Blumenfeld OO: **BGMUT Database of Allelic Variants of Genes Encoding Human Blood Group Antigens**. *Transfus Med Hemother* 2014, **41**(5):346-351.

3. Qian Y, Ziehr JL, Johnson KA: **Alpers disease mutations in human DNA polymerase gamma cause catalytic defects in mitochondrial DNA replication by distinct mechanisms**. *Front Genet* 2015, **6**:135.

4. Sohl CD, Kasiviswanathan R, Copeland WC, Anderson KS: **Mutations in human DNA polymerase gamma confer unique mechanisms of catalytic deficiency that mirror the disease severity in mitochondrial disorder patients**. *Hum Mol Genet* 2013, **22**(6):1074-1085.

5. Taylor RW, Turnbull DM: **Mitochondrial DNA mutations in human disease**. *Nat Rev Genet* 2005, **6**(5):389-402.

6. Stewart JB, Chinnery PF: **The dynamics of mitochondrial DNA heteroplasmy: implications for human health and disease**. *Nat Rev Genet* 2015, **16**(9):530-542.
